# MDBiomarkers: A queryable biomarkers database integrating multiple serum and tissue datasets for Duchenne muscular dystrophy

**DOI:** 10.1177/22143602261458436

**Published:** 2026-06-03

**Authors:** Wangshu Tu, Rebecca A. Tobin, Leenah Abdelrazeq, Kaitey Guite, Cristina Al-Khalili Szigyarto, Roula Tsonaka, Chiara Degan, Yuri E.M. van der Burgt, Jordi Díaz-Manera, Michela Guglieri, Pietro Spitali, Yetrib Hathout, Utkarsh J. Dang

**Affiliations:** 1 6339 Department of Health Sciences, Carleton University, Ottawa, Ontario, Canada; 2 7655 Department of Protein Science, KTH The Royal Institute of Technology, Stockholm, Sweden; 3 4501 Leiden University Medical Center, Leiden, Netherlands; 4 5994 John Walton Muscular Dystrophy Research Centre, Newcastle University and Newcastle Hospitals NHS Foundation Trust, Newcastle, UK; 5 School of Pharmacy and Pharmaceutical Sciences, 14787 Binghamton University, New York, USA

**Keywords:** DMD biomarker database, biomarker website, Shiny application, Duchenne muscular dystrophy, proteomics, mRNA, serum, tissue, reproducibility, biomarker associations

## Abstract

**Background:**

Fit-for-purpose biomarkers are urgently needed in Duchenne muscular dystrophy (DMD). However, biomarker efforts in DMD have traditionally been hampered by a lack of reproducibility due to small sample sizes, confounders such as treatment and age, and discordant findings from different technologies. Moreover, there is no central resource to get an overview of cumulative published evidence. Hence, many researchers often start with new discovery studies, which are time-consuming and costly.

**Objective:**

Build a dynamic, searchable, and easy-to-use biomarker platform for DMD.

**Methods:**

Thousands of molecular (serum proteins and muscle mRNA) markers from multiple studies (28 analyses) were compiled. Findings were obtained from supplemental material of published manuscripts or by following standardized pipelines on available raw data. These findings were annotated with important attributes (e.g., age range, treatment, etc.). Evidence was aggregated around each biomarker’s association with DMD, treatment, age, clinical outcomes, as well as other markers.

**Results:**

The interactive Shiny application on https://www.mdbiomarkers.com provides exportable summaries of serum protein and muscle tissue mRNA findings. This also permits new knowledge to be generated for nuanced meta-analyses, rather than being restricted by a single study’s finding and p-value. A tutorial is provided on the website. This resource is planned to be continually updated with new/additional findings to fulfill the aim of a living biomarker resource.

**Conclusions:**

The resource developed will reduce preparatory time to distill evidence around important biomarker candidates providing summary estimates around individual studies’ effect sizes, help assess cumulative evidence, and help with experimental design of future experiments.

## Introduction

Reproducible biomarker studies are often hindered by a variety of factors, including small sample sizes, poor replication/reproducibility (e.g., %CV) of biomarkers from the same technology in different batches, due to detection limits, and other factors that result in intrinsic variability, discordant findings from different assays/technologies and cohorts.^1^ Duchenne muscular dystrophy (DMD) is a rare, progressive, and fatal neuromuscular condition that occurs due to loss-of-function variants of the *DMD* gene,^2^ and affects approximately 1 in 5,050 male births.^3^ While standards of care exist for DMD, there is no cure, and there is a lot still unknown about its pathophysiology, and the search for better therapeutic targets continues.

While clinical outcome measures such as the time to rise from supine, time to run/walk 10m, time to climb four steps, 6-minute walk distance test, and Northstar Ambulatory Assessment are commonly used as primary endpoints in DMD clinical trials, they have varying reliability metrics^4^ and may not show a clear change in a short period of time. Hence, biomarkers are becoming increasingly attractive for further assessing treatment efficacy, not just safety. In this context, multiple drugs based on exon-skipping^5–8^ and gene therapy^9^ have recently been approved based on accelerated regulatory pathways by showing in the skeletal muscle biopsies production of a truncated, partially functional dystrophin, or expression of micro-dystrophin, respectively. A steroidal drug recently approved relied heavily on biomarker-based de-risking^10^ and demonstration of safety compared to traditional steroids.^11,12^ Hence, there is a large reliance on biomarker-based evidence in DMD.

To establish new biomarkers for specific contexts of use, researchers often start from pure discovery/screening or a limited survey of the literature, without taking full advantage of previously published evidence; this can be wasteful in time and money. This is because, currently, there is no central resource available to get an overview of published evidence around biomarkers in DMD. Similar efforts in other research areas have helped move the scientific literature forward. For example, the Cancer Gene Census^13^ provides a catalogue of genes with substantial published evidence in oncology, MarkerDB^14^ has a dominant focus on genetic and diagnostic markers across different conditions, and the PATRIC database^15,16^(now the Bacterial and Viral Bioinformatics Resource Center) provides genome-scale findings focused on infectious disease research. There are muscle cell- and tissue-specific databases (often not with serum due to a lack of paired muscle and serum samples collected simultaneously) that exist, such as MyoMiner,^17^ which provide co-expression analyses in normal and diseased tissues (including DMD) but do not address crucial questions about prognosis, pharmacodynamics, disease progression, association with serum markers, etc. Hence, in rare neuromuscular diseases, and especially DMD with its unique genotype-phenotype-treatment dynamic, a searchable, central resource is a substantial data accessibility and resource gap.

While the mechanism of action of treatments being investigated is known at a high level, specifics of which molecules are responding to which treatment, whether in muscle (through biopsies; reflecting DMD pathophysiology) or serum (minimally-invasive; reflecting circulating levels), are not completely understood. This can hamper further therapeutic target discovery. Furthermore, while standard biomarkers like Creatine Kinase exist for diagnosis, well-validated and reproducible biomarkers are urgently needed for DMD that also inform on prognosis (how will a patient fare in the future?), pharmacodynamic response (is a biomarker affected by an intervention?), predictive response (will a patient benefit from a drug?), monitoring (reduce trips to clinician; allow for phlebotomy-based remote follow-up), etc., for DMD. Biomarkers have tremendous potential to help clinicians and researchers, but also importantly to alleviate the burden of visiting clinical sites on patients and families, although clinical assessment will continue to provide essential grounding for interpretation of findings, depending on the context.

While large numbers of circulating protein biomarkers have been identified and validated in independent DMD cohorts and across different laboratories,^18–20^ there are many false positives and false negatives in the literature. For example, if we ignore the steroid treated and age range status of the cohort, KEAP1 could be considered high in DMD vs controls,^18^ but multiple steroid-naïve cohorts have shown non-significant decreases in DMD vs controls.^20,21^ Hence, focusing on a single published finding may be riskier due to the potential of it not being reproducible. Above and beyond the factors mentioned above, reproducibility of biomarker research in DMD can be affected by unclear concordance of signals between studies due to a) age (DMD is progressive), b) severity (DMD is known to have large phenotypic variability), c) treatment status (Different corticosteroids are used and new treatments are not implemented in a timely fashion in different cohorts) and d) biosample handling (e.g., temperature considerations). Even among different research efforts using the same platform, findings are not always reproducible unless extremely similar conditions are present during data collection. Translating to real-world adoption of these biomarkers can thus be difficult. In clinics, samples are not always collected similarly, processed in different ways, and assessed variably, which can affect the signal. A recent publication noted a correlation of 0.85 in log2-fold changes between two clinical trial-based cohorts with a similar age range using Somascan technology, a huge jump from a correlation of 0.31 seen before in more dissimilar (natural history) cohorts using the same Somascan panel.^21^ Similarly, another recent paper showed good correlation between two different geographical and health system cohorts on the same Somalogic assay.^22^ However, strict control and large similarity across cohorts are not always possible.

It would be ideal if a dynamic platform allowed researchers to quickly identify which markers already have a large body of evidence for a specific question of interest, across different assays. A resource that allows for easy curation of findings across different clinical (human) cohorts to establish overall patterns is crucially needed. With such a resource, patterns can be aggregated and summarized across studies, no longer restricted to a single fold change or p-value from a single study. Motivated by this, our objective was to build an online, searchable, living (more datasets will continue to be added), open-access database that compiles evidence on biomarkers (starting with serum proteins and tissue mRNAs) for multiple neuromuscular diseases, starting with DMD. Considering their importance, data are also curated around association with DMD, treatment, serum vs muscle comparative levels to healthy controls, age, clinical outcomes, etc.

## Methodology

This study was cleared by the Research Ethics Board at Carleton University.

### Focus on serum datasets

While muscle tissue-based transcriptomic (mRNA expression profiling, miRNA and protein expression profiling) markers have historically been used to understand DMD histology and severity, there is a movement towards minimally invasive serum biomarkers. As well, given the young age range in which there is the greatest potential to alter the future course of DMD, and the target of many trials, we focused more on minimally invasive serum or plasma proteins at a young age. Given that serum activity may or may not reflect muscle activity, we also connected findings on these circulating proteins to muscle tissue-based mRNAs from other studies.

Many technologies are available in the literature, including MSD ELISA, RT PCR, Somascan®, mass spectrometry, antibody-based Luminex assays, and others. To build a database with an abundance of possibly DMD relevant biomarkers, we sought out manuscripts that were published on large-scale (studying hundreds or thousands of targets) biomarker quantification efforts.

### Publications reporting on serum and muscle findings

Proteins from multiple serum and mRNA/gene expression from muscle (biopsies; Affymetrix assays) biomarker publications were compiled with a focus on (minimally invasive) serum markers. The findings were obtained from supplemental material of published papers, or by applying standardized processing pipelines (log2 transformation, normalization, filtering) using state of the art models provided by empirical Bayes-based moderated tests from *limma*,^23^ correlation-based clustering in available treatment-naïve Somascan datasets^21^ using *WGCNA*,^24^ etc., when raw data were available; the latter was done when both raw data and supplemental material were available to provide findings from the same standardized analysis pipelines. The evidence was aggregated around each biomarker’s association with DMD, treatment, age, clinical outcomes, and other biomarkers. These findings are annotated with attributes (e.g., age range, treatment type) that can be easily filtered or inspected.

### Multiple IDs and multiple targets per marker

Technologies provide quantification of different targets, unique isoforms, etc., that are linked to the same Uniprot ID. Furthermore, noting that multiple ID systems (UniProt, Entrez, ENSEMBL) are used in the literature, for consistency, we consider a biomarker to be a marker associated with a UniProt ID. This means that fragments which originate from the same gene (e.g., many fragments of C3, including C3a, C3b, C3d, iC3b, and C3adesarg), including all proteoforms, are grouped under one UniProt ID in our compilation. When data were available (not all studies provided this in their Supplemental Material) to discern between these targets, e.g., from Somascan, this was retained for comparison across different studies, but aggregated under the same UniProt ID. Similarly, when multiple probes (e.g., Somalogic aptamers, Affymetrix probes) are available per target, all individual signals were retained and provided (rather than aggregating them together in some form) given that some of the aptamers, or probes may be relatively more or less effective in terms of quantification of signal given the properties of the serum sample and the biomarker target. Finally, if any reported findings had multiple UniProt IDs associated with a single biomarker (e.g., IL12B-IL23A and TSHB-CGA heterodimers from Somascan data), the single record that has multiple UniProt IDs is split into multiple UniProt observations and allowed to be searched for accordingly.

### Published data/findings preprocessing and structure

For each published dataset, a preprocessing pipeline was followed, including standardizing variable names, checking biomarker identifications (e.g., linking Affy probe ID to updated gene information using g:Profiler^25^ g:Convert), retaining target/probe name, converting fold ratio to standardized fold changes (FC) and log2 fold changes as needed. If a published study with a large number of targets analyzed only provided raw p-values, we corrected the p-values using false discovery rate correction. Across different studies, we used up to 4 variables altogether to aggregate findings, and any of the following can be used to search for a marker of interest: UniProt ID, Entrez Gene Symbol, and Entrez Gene ID (in rare cases, we also used the Target short name).

### Data joining to biological annotations

Access to one-click biomarker-specific external links to *Uniprot (*https://www.Uniprot.org*), QuickGO* (https://www.ebi.ac.uk/QuickGO/*)*^26^*, and The Human Protein Atlas (*https://www.proteinatlas.org*)*^27^ were also built in. Similarly, known involvement in disease connected to the OMIM database was also provided.

### Technical aspects (website and interactive application)

The website is created with Quarto^28^: an open-source scientific and technical publishing system. The interactive application is built with the s*hiny*^29^ and *shinydashboard*^30^ packages in *R*^31^ and embedded into a Quarto webpage. All the visualizations are created via R packages: *ggplot2*^32^*, plotly*^33^*, DT*^34^*, corrplot*^35^*, bslib*^36^*, bsicons*,^37^ and *fontawesome*.^38^

## Results

### Website and tutorial

**Figure 1.**
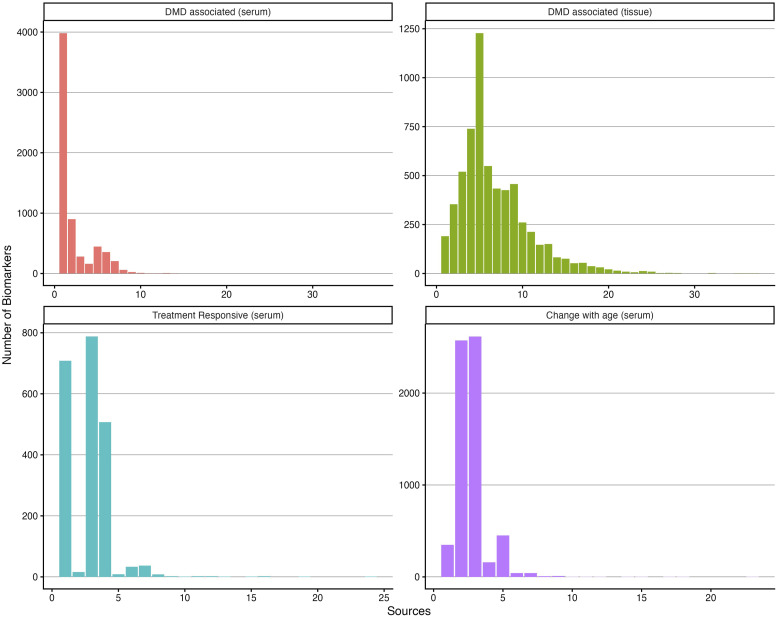
Summary of datapoints currently available. From top-left to bottom-right, this provides histograms of how many findings (x-axis) are currently available for how many biomarker targets (y-axis). The top two subpanels show the DMD-associated serum subpanel summarizing data on serum studies in DMD (including different aptamers for the same target) vs healthy controls, and the DMD-associated tissue subpanel summarizing data on mRNA studies from muscle tissue biopsies for each target (including different probes). The last two subpanels provide aggregate numbers on those serum targets that respond to therapies, and data points available corresponding to them; and those studies that summarized longitudinal association with age in serum.

More than 6000 biomarker targets (Figure 1) have been compiled from 28 analyses, with a focus on circulating serum proteins^18–21,39–41^ and corresponding tissue-based mRNAs.^42–47^ Findings from Somascan**®**, tandem mass tag (TMT) mass spectrometry, Affymetrix**®**, array microarray, and RT-PCR platforms are incorporated into the biomarker app. SomaScan uses aptamers to simultaneously quantify thousands of proteins in a sample. Each aptamer is tagged with a unique short DNA sequence and binds specifically to its target protein. After binding, the DNA sequences corresponding to each aptamer are quantified using a high-throughput microarray. Protein abundances are reported as relative fluorescence units (RFU) derived from the microarray signal. A similar depth of protein identifications but with lower sample throughput can be obtained with label-free mass spectrometry. TMT-based mass spectrometry uses isobaric tags to label tryptic peptides generated from proteolyzed proteins. Various TMT kits enable the simultaneous analysis of 6 to 18 samples in a single run. Each tag contains a reporter ion with a specific mass that is detected by mass spectrometry; the intensity of the reporter ion reflects the relative abundance of the peptide in the sample labeled with that tag. The relative peptide intensities are then aggregated to determine the relative abundances of the corresponding proteins. However, each method has its own advantages and disadvantages. SomaScan offers high multiplexing and greater sensitivity compared to TMT but is limited to detecting and measuring aptamer-targeted proteins. In contrast, TMT-MS-based quantitative proteomics is more labor-intensive and time-consuming, yet it is highly specific and capable of discovering novel biomarkers. Overall, highly multiplexed, quantitative proteomic profiling in a single run can be obtained by using tandem mass tag (TMT) labels or multiple reaction monitoring (MRM) strategies, albeit that the latter is limited to 10s of proteins and cannot multiplex hundreds or even thousands of proteins like Somascan.

The website, https://www.mdbiomarkers.com, and the embedded interactive Shiny application prioritize an intuitive user experience; moreover, a tutorial and frequently asked questions (FAQ) page are provided.

A clickable bibliography of manuscripts is also provided for the researcher user to easily cite the original paper of a finding of interest, along with some key important attributes (age, treatment status, sample size, assay technology). Similarly, tools used in the data pre-processing/curation pipeline, and citations for all tools used to build the website, interactive application, and visualizations are provided.

### Interactive Shiny application: Overview and biomarker-specific view

**Figure 2.**
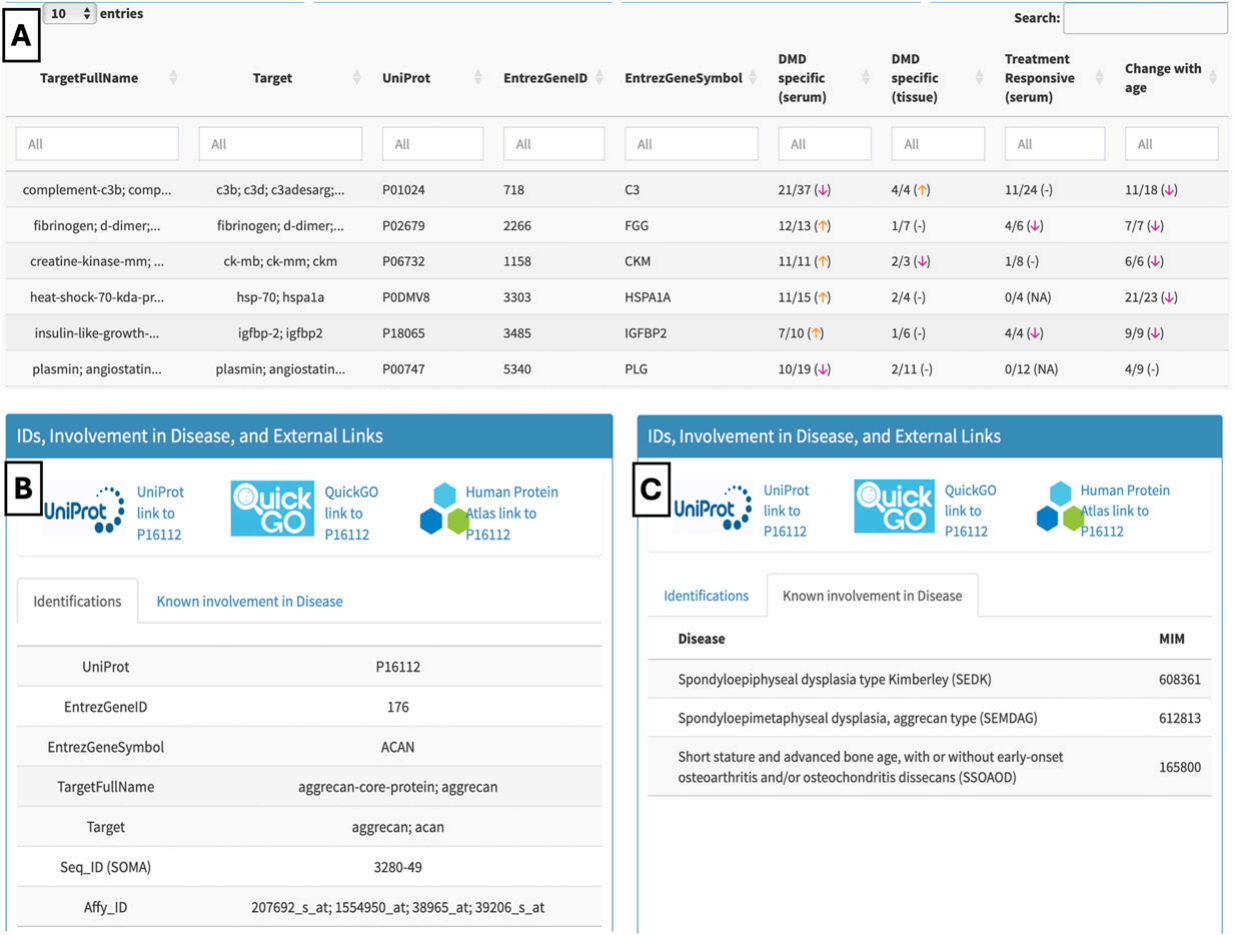
(A) shows an overview table in the interactive app, allowing for a quick search of the biomarker of interest and getting an overall summary of the findings (fraction of significant findings) for that biomarker. (B) shows a biomarker-specific page that can be opened by searching for and clicking on ACAN as an example. Biomarker IDs as well as one-click external links to Uniprot, QuickGO, and Human Protein Atlas are provided. (C) shows known involvement in disease (for ACAN here). The UniProt, QuickGO, and Human Protein Atlas logos are the property of their respective organizations and are used for illustrative purposes only.

On the overview page (Figure 2) in the interactive Shiny application, the user can search for a biomarker target based on the target name, target short name/ID, the UniProt ID, Entrez Gene ID, and Entrez Gene symbol. Using the Boolean OR symbol “|” in any of the filter/search boxes under the column heading (e.g., P01024|P06732 under UniProt, or FCER2|CKM under EntrezGeneSymbol), the overview page can be filtered to show multiple biomarkers for overall comparison. Summary columns provide information on the number of total findings (including multiple proteoforms or multiple probes) and the number of statistically significant findings for each biomarker for a) DMD vs healthy control altered levels (serum and tissue), b) treatment responsiveness (serum), and c) change with age. This facilitates quick comparison between different biomarker targets. If available, the typical directionality (up or down arrow) of finding is also provided. Users can have a quick overview of how many times that biomarker has been studied and the fraction of records that show a significant relationship and direction.

In those cases where there are no fragments or isoforms of the protein in the merged dataset, the fractions provided in the table reflect the consistency of getting a statistically significant finding across different studies based on adjusted p-values. However, in other cases, for example, where there are different targets/fragments under a single UniProt ID, some fragments may have strong associations and others may not, but these results are still combined into a single fraction; hence, we do not recommend making conclusions based on the overview table only.

Rows of information on specific biomarkers on the overview webpage are clickable, opening up a view with detailed, curated information that provides details on the questions of interest with available information (Table 1). In this more biomarker-specific view, all the identification information is provided, along with Somascan IDs and Affymetrix probe IDs, if relevant. Multiple sub-panels capture information relevant to the questions of interest, for which we focused on aggregating findings across studies (Table 1). Available cumulative information is provided as tables or figures capturing fold changes, raw and multiple testing corrected p-values, and other useful statistical measures (correlations, or sign of association when the measure is not directly interpretable without additional information on data like a regression coefficient) seen across studies.

**Table 1.** Types of questions answered via the biomarker resource.

Category	Specific questions answered	Outputs available	Relevance/Importance
Disease-associated serum biomarker	1. Is a biomarker elevated or depressed in the serum of patients with DMD compared to healthy controls?	Directionality, fold change, raw p-value, adjusted p-value	Drug target screening, diagnosis, monitoring
Disease-associated tissue biomarker	2. Is it elevated or depressed in the muscle tissue of patients with DMD compared to healthy controls?	Directionality, fold change, raw p-value, adjusted p-value	Drug target screening, diagnosis, monitoring
Pharmacodynamic* response	3. Does this biomarker respond to (standard of care for DMD) treatment?	Directionality, fold change, raw p-value, adjusted p-value	Drug target screening, monitoring, predictive, monitoring, clinical trial design, prognostic
Change over time	4. Does this biomarker change with age in DMD? How does this compare to what’s known in healthy controls?	Directionality, raw p-value, adjusted p-value	Monitoring, predictive, clinical trial design, prognostic
Protein-protein correlations	5. Which other biomarkers respond similarly in DMD serum to a specific biomarker?	Association strength (biweight midcorrelation)	Building assays for proteins not possible on certain technologies, understanding pathophysiology, pathways, and networks.
Association with clinical outcomes	6. Does this biomarker associate with clinical outcomes?	Association strength (Spearman correlation)	Monitoring, acute change, remote trials and studies, predictive clinical trial design, prognostic, response to treatment

*Currently, only steroid response datasets are included, but response to other therapies will be added as they become available in the literature.

The tutorial walks through each subpanel of information. The FAQ provides important considerations such as when conducting a meta-analysis like overall summarization is appropriate (we recommend doing this at as specific level as possible, e.g., specific target (aptamer, probe), while controlling other factors like age, treatment status, technology of biomarker quantification), why there is a discrepancy on significance of finding among similar studies, and how it is important to look at the consistency of directionality, fold change, and p-values across different studies. Among other considerations discussed, it also discusses how confounding due to differing treatment (currently, steroid) status can lead to different findings in different cohorts, e.g., suggesting differences in biomarker levels between DMD and healthy controls in 1 study but not the other.

### Outputs available

Each biomarker-specific page contains 3 one-click links to UniProt, QuickGO, and the Human Protein Atlas website, which provide more biological annotation for that biomarker, as well as a listing of all known involvement in diseases and the MIM ID for the diseases.

Fold changes and p-values are available for questions 1 through 3, directionality of change and p-value for question 4, and Spearman correlation coefficients for questions 5 and 6 in Table 1. Some biomarkers will have more findings for a specific question compared to others, for example, most biomarker targets have between 2 to 9 sources of findings compiled for question 1 (Figure 1). Many of these subpanels allow for filtering of findings by specific target name as well as the age range of DMD boys in the dataset.

Both plots and tables are provided capturing the same information (Figure 2), and it is possible to export these as outputs (image, CSV, or Excel file) to the user’s computer. A target-specific report including the information aggregated on the Shiny app for that target can also be exported directly to the user’s computer. Fold changes and p-values are provided via volcano plots (axes are log2 fold change and -log10 of adjusted p-values), in which the datapoints have tooltips on mouse hover that provide for single finding datapoints the citation, and based on relevance, the number of DMD samples, the number of healthy control samples, age range of subjects whose samples were used, their treatment status, duration of treatment, assay technology, fold change, and p-value. A colour code is used to indicate the statistical significance, while the different shapes of the data points indicate (orange=statistically significant at alpha=0.05 and with multiple testing correction; blue=non-significant using complementary criteria) the assay technology used.

For displaying protein-protein associations in serum, the correlation plot provides the top 10 most correlated biomarkers from the cluster to which the protein of interest was assigned to along with the Spearman correlations. For association with age, a lollipop plot is provided, which indicates the directionality of change over time (age).^18^ Above the lollipop plot, we also have a summary table indicating the findings from Liu (2017),^40^ which investigated the stability of these proteins from plasma samples (using TMT 10plex) in young, healthy controls. Finally, a correlation plot shows the correlation between biomarkers and available clinical outcomes (if an outcome is missing, a question mark is shown).

### New knowledge created

Having correlation-based clustering results on serum biomarkers can help with experimental design when there are constraints. For example, if two biomarkers are highly correlated in Somascan data (and this has previously been validated orthogonally), and a researcher wants to use an absolute technique on one of these biomarkers, but the preferred assay is not available for that target, then an alternative could be to use the absolute quantification technique on the other highly correlated biomarker.

Nuanced meta-analyses for a specific target are also enabled through this biomarker resource. Different papers could have different findings; for this, there could be many reasons, e.g., different age range, different treatment status (corticosteroid-naive or corticosteroid-treated) or regimen (daily treatment vs intermittent, etc.), different technology, or less statistical power of the statistical technique used. When looking at the cumulative knowledge, we recommend not focusing on the p-value alone but also looking at the fold changes and the directionality (paying close attention to probe, age, treatment status, etc.). If the fold changes across multiple studies (especially with the same technology) with similar cohort characteristics are generally consistent, a non-significant p-value in a specific study likely reflects a lack of power due to small sample size (often but not always due to false discovery rate multiple testing correction). Similarly, an extreme fold change seen in one study doesn’t necessarily mean a real difference; it could be a Type 1 error.

Hence, this new knowledge can be created with this biomarker resource. For example, we can evaluate findings from 7 different datasets on transketolase (TKT; P29401), and notice that while all 6/7 fold changes were significant, 5 of these were positive fold changes ranging between 1.49 to 3.94 vs healthy controls (Somascan results),^18,20,21,41^ one a significant negative fold change (-1.82; TMT result^19^), and the non-significant fold change was 1.81 with an adjusted p-value of 0.074^18^ from a Somascan comparison. This suggests that the non-significant (based on adjusted p-value) Somascan finding was a likely Type 2 error (false negative) and that the “predicted” direction is positive for the Somascan target. The TMT difference may be due to a different target being captured between TMT vs Somascan.

We identified biomarker targets where there is a majority signal (similar directionality in the majority of significant findings) in both serum and tissue studies across findings from different studies. For these identified markers, Supplemental Table 1 provides whether these markers were increased or decreased in DMD as compared to controls in tissue and serum. The largest category of such markers with majority signal was increased both in serum and tissue. While not from a paired study of serum proteins and muscle mRNAs, by combining results across different studies, this may still help understand whether serum protein fold changes represent the same directionality as in muscle or not.

### Example case studies

**Figure 3.**
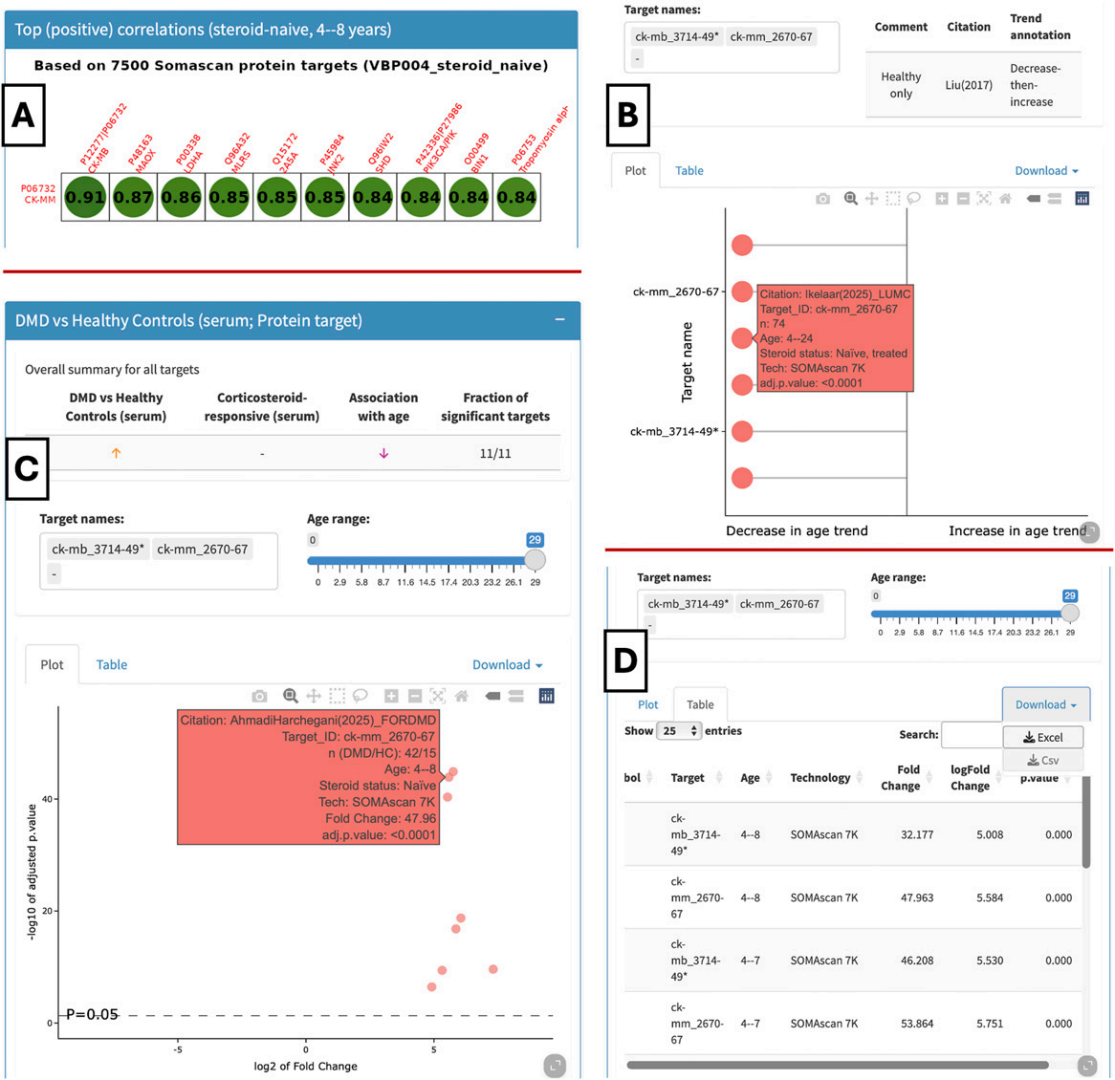
The subpanels use CKM as an example. (A) shows top biomarker-biomarker associations (CKM associated strongly with CK-MB, MAOX, LDHA, etc.). (B) shows the trend with age seen in DMD studies, as well as whether information is available about the longitudinal trajectory in healthy controls. (C) shows DMD vs healthy controls from a variety of datasets (both CKM and CKB are targets aggregated together here). The volcano plot shows 11 sources of data for this target, all of which are significant and increased in DMD vs controls (note 3 are not visible when the figure was made due to the on-hover tooltip). The plot can be switched to a table (D) and exported. Similar plots are available for treatment response in serum, as well as differences from healthy controls in mRNA tissue. All datapoints in the volcano or lollipop plots have on-hover tooltips that provide attributes of the contributing dataset and finding.

We provide some brief examples of summaries that can be obtained by searching for a few proteins on the interactive application. Creatine Kinase m type (CKM; P06732) is a classic, screening/diagnostic biomarker for many muscular diseases, including DMD and in newborn screening. It rises sharply in the early stages of the disease due to sarcolemma instability and the leakage of muscle enzymes into the bloodstream. Previous research has demonstrated that CKM and other “CKM-like” muscle injury biomarkers are significantly elevated in young, untreated boys with DMD.^20^ These biomarkers often decline over time as muscle mass decreases, making them valuable indicators of early muscle damage and potential pharmacodynamic response for certain drugs like gene therapy. Based on the MIM database, CKM does not have a polymorphism associated with a known disease. The Human Protein Atlas shows CKM to be enriched in skeletal myocytes. The Shiny application shows 11 findings (Figure 3) for DMD vs healthy controls in serum (including aptamers for both CK-MB and CK-MM), all of which have positive statistically significant fold changes^18,19,21,41^; this is true both in treatment-naïve and treated DMD comparisons to controls. It shows that the TMT finding was the smallest in terms of fold change.^19^ In terms of protein-protein interactions, LDHA was found to have a very strong correlation (0.81) in serum with CKM. There is unclear evidence as to whether it is differentially regulated in tissue vs. healthy controls, and whether it responds to treatment in.^42,44,47^ CKM has only weak correlations with clinical outcomes in treatment-naïve boys at a young age. Finally, in healthy boys’ plasma, it is known to first decrease then increase^40^; whereas, in DMD, it shows a clear decrease over time across multiple analyses^18,22^ (which is consistent with what’s well known about CKM in DMD).

Leptin (P41159) is a fat-associated biomarker, which may be associated with subcutaneous fat, especially in steroid-treated patients. Based on the MIM database, LEP is associated with Leptin deficiency (LEPD; MIM: 614962). The Human Protein Atlas shows LEP to be enriched in adipose tissue. The Shiny application shows 7 findings for DMD vs healthy controls in serum, 4 of which have negative statistically significant fold changes, and the other 3 have non-significant positive fold changes. The ambiguity seems to result from steroid exposure; all 4 findings are from younger and steroid-naïve cohorts^20,21^ when intramuscular fat is low, meanwhile all the 3 non-significant positive fold changes were from older but also steroid-treated cohorts^18,41^ (which can be seen by hovering on the datapoints in the volcano plot). No protein-protein interactions were greater than 0.54, ignoring the other somamer/aptamer also targeting leptin. There is unclear evidence as to whether it is differentially regulated in tissue vs. healthy controls (5 non-significant findings), but leptin seems to increase on steroid-treatment in serum (more significant findings for prednisone; more studies needed on deflazacort). Finally, in healthy boys’ plasma, it is known to increase; similarly, in DMD, it shows an increase over time across multiple analyses (which is consistent with steroid treatment as well). In comparison, another fat biomarker, FABP4, has clear findings on decrease in DMD serum vs healthy controls,^21^ increase over time in DMD^18,22^ (vs flat in healthy boys plasma), likely increased in DMD tissue (3/8 significant findings but 3 other findings with a similar fold change but non-significant adjusted p-values perhaps due to low sample size), and unclear findings regarding prednisone or deflazacort (1 finding decreased) response.

### Living website

The website is planned to be updated moving forward with additional researcher-published or submitted datasets to be incorporated into the biomarker resource website. This will involve using aggregate findings published as Supplemental Material and annotating with important attributes (treatment status, age of patients for whom samples were included, etc.) or running minimal quality checks on user-submitted data with automated pipelines (i.e., using internal SOPs to allow semi-automatic aggregation of new evidence).

## Discussion

We have released a publicly available, open-access, searchable, and filterable database of >3500 biomarkers from DMD patients with 28 studies/analyses compiled. This can be accessed via an interactive Shiny application hosted on https://www.mdbiomarkers.com. DOI-based one-click external links and references are provided to the original publications from which the data/findings were obtained for easy citation by users. This knowledge database and repository/tool provides summary estimates around individual studies’ effect sizes and helps assess cumulative evidence, not possible with a single study’s findings. Support for this resource is provided for researcher users via a tutorial and FAQ.

To generate this compilation, the totality of evidence was aggregated around each biomarker’s association with DMD in serum vs. tissue, protein-protein interactions, known involvement in other diseases, treatment, age, and clinical outcomes, among others. A detailed, biomarker-specific view with functionality to export evidence around a biomarker or download a report was generated. This website was designed with the user (researcher’s) experience in mind, was tested by international DMD researchers, and their feedback was incorporated.

Currently, the biomarker resource is focused on DMD and serum biomarkers, although it provides integration with muscle datasets as well. This biomarker resource should facilitate quick comparison of new findings from a research lab to published findings, new knowledge in terms of nuanced meta-analyses for a specific protein target, reduce preparatory time, and aid with the design of future experiments including orthogonal validation, allow for data mining of biomarker patterns, and help with building regulatory submission packages for “fit for purpose” contexts. The goal is for this website to be continuously updated with additional findings to make it a living resource.

It’s important to note that the introduction of new treatment options into the standard of care is not harmonized worldwide and largely depends on regulatory authorities’ recommendations and reimbursement strategies. In this context, the availability of a dynamic and accessible biomarker platform for DMD will become even more important as confounding factors in small cohort studies will further increase.

It is worth discussing two points of interest discovered while assembling this resource. While any two datasets may have different findings for a biomarker, often, using aggregated evidence from multiple (>2) datasets allows for easier understanding of a biomarker’s importance (while keeping nuances about age, treatment status, etc., in mind). Having said that, while evidence on biomarker-clinical outcome correlations is included in the resource due to its importance, we also found this to be a question with poor reproducibility across studies. This is not surprising; correlations and regression modeling require larger sample sizes than usually seen in DMD datasets (especially compared to a differential analysis, e.g., comparing DMD vs controls), convenience samples are often not comparable in patient characteristics, reliability of both outcomes^4^ and biomarkers affects the association, and it’s possible that certain narrower age ranges are not conducive to this question. Hence, more work is needed in this area where a more consistent signal may start to emerge with more datasets and multivariate modeling. Also, note that while currently biomarker-clinical outcome correlations are only included and summarized in a short age range prior to treatment, this will be expanded to include on treatment as more datasets get published. The other important point to note is that some datasets published as Supplemental Material often did not provide enough information on the identification of the protein or probe used. Our recommendation to researchers is to include as much biological identification annotation as possible, including Uniprot ID, probe ID (if relevant), target, target name, Entrez gene symbol, etc., so that a proper comparison can be conducted against findings from other publications.

A current limitation is the preponderance of data from larger multiplex studies using the Somascan platform, TMT, etc. There are data on other notable technologies currently missing, e.g., Olink, ELISA, label-free MS-based proteomics (including MRM and Data-Independent Acquisition), etc. Paired serum-muscle tissue biomarker data were also not found in the literature, so different cohorts are currently integrated in the database. As researchers make these available, they are planned to be incorporated into the database. The website provides contact information for other researchers to reach out to have their biomarker findings hosted on the database. Furthermore, newly published findings from large biomarker studies, when available, will be uploaded to the website twice yearly. Inclusion criteria for such datasets includes human samples, treatment response to approved therapeutic drugs, available attributes regarding the source muscular dystrophy samples (sample size, cohort age range, treatment status, etc.), and datasets with sufficient methodological detail allowing for transparent display of metadata to allow users to assess study quality. More features are planned, including incorporating findings from other muscular dystrophies, other biomarker types (e.g., snRNAseq,^48^ metabolites, miRNAs), biomarker response to other treatments like exon skipping, gene therapy, as literature becomes available on these, etc.

## Conclusion

We have released an openly available website https://www.mdbiomarkers.com that hosts a free to use available electronic database of biomarkers, curating thousands of findings from multiple serum and tissue datasets in DMD. This is a searchable, living resource for researchers to quickly get an overview of current cumulative evidence around biomarkers (proteins in serum, mRNA in tissue) in DMD. The resource will reduce the time spent assessing evidence around important biomarker candidates, provide summary estimates of individual studies’ effect sizes, and assist in designing future experiments.

## Supplemental material

Supplemental material - MDBiomarkers: A queryable biomarkers database integrating multiple serum and tissue datasets for duchenne muscular dystrophy Supplemental material for MDBiomarkers: A queryable biomarkers database integrating multiple serum and tissue datasets for duchenne muscular dystrophy by Wangshu Tu, Rebecca A. Tobin, Leenah Abdelrazeq, Kaitey Guite, Cristina Al-Khalili Szigyarto, Roula Tsonaka, Chiara Degan, Yuri E.M. van der Burgt, Jordi Díaz-Manera, Michela Guglieri, Pietro Spitali, Yetrib Hathout, Utkarsh J. Dang in Journal of Neuromuscular Diseases

## Data Availability

Most of the data mentioned in this article are previously published and available online.*
